# Self-Harm in Aviation Medicine—A Complex Challenge During a Pandemic

**DOI:** 10.3389/fpubh.2021.681618

**Published:** 2021-08-02

**Authors:** Alpo Vuorio, Robert Bor

**Affiliations:** ^1^Department of Forensic Medicine, University of Helsinki, Turku, Finland; ^2^Mehiläinen Airport Health Center, Vantaa, Finland; ^3^Center for Aviation Psychology, London, United Kingdom; ^4^Royal Free Hospital, London, United Kingdom

**Keywords:** aviation, pilot, mental health, self-harm, suicide, COVID-19

## Introduction

Several mental health problems are associated with self-harm. These include borderline personality disorder, depression, bipolar disorder, schizophrenia, and drug and alcohol-use disorders (1). The management of these disorders is not the scope of this paper. Rather, we discuss how the risk assessment for future self-harm and/or suicide is carried out in the aeromedical context. One challenge is that many individuals do not receive an adequate and timely psychological assessment at hospitals and in healthcare in general (2). In these cases, it is possible that aeromedical examiners (AME) or aviation psychologists are among the first professionals who carry out the risk assessment of aircrew. This task is specialized and demanding and to date, there is a paucity of specific guidance or articles available on how this should be conducted. The importance of self-harm in safety-critical work settings has not been extensively studied (3). The COVID-19 pandemic has had a significant impact on the aviation industry leading to job loss, furlough, disruption to careers and training trajectories and uncertainties among airline employees. There is also evidence that COVID-19 disease itself can cause neurological and psychiatric morbidity (4).

Examples of presentations of self-harm are self-poisoning with medication or self-injury by cutting (5). Excessive alcohol consumption or accidental harm to oneself are usually not included in self-harm. Self-harm is not a disorder, but it is commonly associated with several definable mental health problems and conditions including personality disorders, depression and bipolar disorder, which may compromise aviation safety (6, 7). Those patients who self-harm have a 50–100-fold higher likelihood of dying by suicide in 1 year follow-up compared to those individuals without any self-harm acts (1). Recent research into the genetic etiology of non-suicidal and suicidal self-harm was published (8). Researchers found seven genes associated with self-harm ideation and four genes with self-harm behavior. Despite preliminary findings clearly more research of genetic etiology of self-harm needs to be carried out. The relationship between self-harm and suicide is complex because people often switch methods of self-harm (9). Several risk-assessment scales have been evaluated to determine whether they may predict suicide risk following self-harm. Recent meta-analysis showed that use of the scales is not practical as predictors (10) and instead comprehensive psychosocial assessment and individualized risk analysis could be useful practices.

## Impact of COVID-19

While there are concerns that a pandemic may have an impact on increasing suicide risk (11, 12), the direct impact of a pandemic on self-harm is complex, uncertain and still under investigation (13, 14). It is evident that emotional distress increases during pandemics (15). Furthermore, those being treated for existing mental health disorders may experience a worsening of their condition during pandemics (16). Additionally, undiagnosed mental health conditions could worsen during a pandemic and therefore become clinically relevant. To date, there is no clear indication that self-harm rates have increased during the pandemic, at least in UK. It has been also shown that the impact is dynamic and may change in the course of different phases of the pandemic (17, 18). It is also possible that when the pandemic crisis finally ends, self-harm and suicide rates may increase (19).

From the aviation health and safety point of view, self-harm is a complex issue, and the guidance provided for assessment is limited (20). The term self-harm is referred to as an act of self-poisoning or self-injury carried out by the person itself (21). There are different estimates of the prevalence of self-harm. In larger population-based studies in UK, the self-reported lifetime prevalence of non-suicidal self-harm increased from 2.4% (95% CI 2.0–2.8) in 2000, to 6.4% (5.8–7.2) in 2014. It is estimated, based on literature reviews, that rates of community-based self-harm (300–1,100/100,000/year) are much higher compared to hospital-treated self-harm (2.6–542/100,000/year) (22). The range of self-harm presentations is wide, and many presentations will have a benign outcome.

The difficulty in assessing the prevalence of self-harm is compounded by the fact that use of healthcare has decreased during pandemic. The real-time University College London COVID-19 Social Study (https://www.covidsocialstudy.org) shows in the latest report published on 01 March 2021 that the incidence of self-harm has remained stable being about 4% in the course of the pandemics (30 March 2020–01 March 2021). Interestingly, self-harm remains higher among young adults. Regarding airline pilot mental health, there is a concern that the risk of suicide may increase due to the combination of unemployment, skills-fade and pandemic-caused stress including profound lifestyle changes (23). No supporting data is yet available regarding increased suicidality or self-harm among unemployed pilots. There is, however, the possibility that sudden major changes in society may have impact on pilots' mental health. We have shown that after the 9/11 terrorists attacks in New York, the risk of aircraft assisted suicide significantly increased for a 1 year period (24). At the societal level, both risk factors arising from a pandemic and additionally protective factors that can be actively influenced can be identified (19). Table 1 shows these factors from an aviation health perspective.

**Table 1:** Factors related to aero-medical mental healthcare during pandemic.

**Factors**	**Society/airline**	**Impact**
Risk factors for psychological distress	Difficulties in accessing aero-medical healthcare	Discontinuation of periodical medical examinations
	Impact of over-negative news related to aviation	An over-negative perception of one's own future
	Stigma associated with the use of healthcare services	Fear of the negative impact of health issues on current job or job search
Protective factors for psychological distress	Government funds to improve healthcare	Positive impact but need for aero-medical healthcare
	Strengthening mental healthcare systems	Positive impact but need for aero-medical mental healthcare
	Continuation and strengthening aviation occupational health services	Very positive impact

*Modified from (19)*.

## Clinical Evaluation of Self-Harm

As mentioned above, several mental disorders are associated with self-harm including borderline personality disorder, depression, bipolar disorder, schizophrenia, and drug and alcohol-use disorders (1). Differential diagnosis is demanding and requires the expertise of an experienced psychiatrist and psychologist ideally one familiar with aviation. Questionnaires such as the Patient Health Questionnaire (PHQ9) (25) and the seven-item Generalized Anxiety Disorder Scale (GAD-7) (26) can be utilized as a first steps of differential diagnosis, although there may be some limitations when using these with pilots who may try to manage impressions of them (27).

Aeromedical examiners (AME) may meet and assess young applicants who are on the way to become aviation professionals after leaving school (28). The history of self-harm in adolescence is in many cases revealed when pilots first apply for medical fitness licenses. Moran et al. (29) studied the natural history of self-harm during the transition from adolescence to young adulthood among 1,802 young people in Australia. The authors showed that most self-harming behavior in adolescents resolves spontaneously. In adolescents, 149 (8%; 95% CI 7.0–9.5) of the participants reported self-harm, but in follow-up 122 participants who reported self-harm during adolescence reported no further self-harm. Symptoms of depression and anxiety in adolescents were associated with self-harm continuity in young adulthood. Prognosis is clearly different with repeated self-harm occurrence for young adults (30). In the study involving 2,559 12–25 year old participants carried out in UK from 2004 to 2007, approximately one fifth of 12–25-year-olds presented with repeat self-harm episodes in an average 18 months of follow-up.

## Guidance for Self-Harm Assessment by Aviation Authorities

What guidance is given to AMEs by different aviation authorities? To map the current situation of guidance provided by different aviation authorities: US Federal Aviation Administration (FAA) (31), European Aviation Safety Authority (EASA) (20), Transport Canada (32) and International Civil Aviation Authority and International Civil Aviation Organization (ICAO) (33) websites were searched to find out their assessing guidance regarding self-harm. This search revealed that only EASA provided guidance for self-harm history-related eligibility assessment (20). EASA recommends detailed individual-based risk analysis taking account both the history as well as current psychiatric and/or psychological concerns. Additionally, EASA highlights the possibility of conducting a neuropsychological assessment. No specific clinical risk assessment scale is recommended.

## Factors Suggesting Further Evaluation Among Pilots

When assessing the risk of a pilot who has previously self-harmed, to a new self-harm incident or episode, there may be only limited applicability to use of research data achieved from the general population. The clinician should be aware of common factors predisposing to self-harm. In the recent Australian cohort study involving 1,962 individuals aged 12–30 years who visited a mental health clinic, researchers examined the predictors of within 6 months repeated self-harm (34). The three strongest predictors were a history of self-harm, younger age and social and occupational functioning measured by low value in the Social and Occupational Functioning Assessment Scale (SOFAS) (35).

In the aviation medicine and psychology context, clinicians' assessment for self-harm is important for three main reasons. Firstly, self-harm can potentially affect flight safety. Secondly, self-harm may be related to an underlying mental health condition. Finally, in extreme situations, the individual may lack insight into their mental state and the severity thereof, making assessment all the more imperative by a trained clinician.

The major challenge regarding pilots is that they may not necessarily report self-harm intent or behavior to the AME for fear that it could jeopardize their ability to work (36, 37). The AME is inevitably not undertaking a “gold standard” mental state assessment, but an abbreviated evaluation, which could be enhanced by an understanding of the workplace demands, but equally could be impaired by the reluctance of the pilot disclosure concerns with potential job impact. We have previously shown that when analyzing approximately 200 hundred fatal aircraft accidents that occurred in the United States during the year 2015, in approximately 5% of these cases the pilot had either a taken medication or suffered from disease which was associated with the fatal aircraft accident and was not reported to AME (37). While there is no simple solution for this challenge, the well-structured duty of notification process is needed.

Mental health and risk assessment takes several forms. It should review past mental health problems, particularly given the link between these and lifetime risk of psychiatric ill-health. There are several contexts in which the assessment is made. The first is in the context of pilot training where cadet pilots form a close relationship with their flying instructors. It is more common in this context for emerging or existing mental health problems to be detected. The second is the aviation medical examiner's formal assessment of the patient. This will likely take into account both their clinical presentation when meeting them, a review of their general health history and also, where accessible, a view of their full medical record. Issues pertaining to psychological conditions may be documented in the candidate's health record. Collateral information may also be obtained from third party sources such as counselors or therapists that the individual may have seen in the past or from national database records such as police records detailing antisocial personality or incidents of antisocial behavior or driving under the influence of alcohol and/or drugs. Whilst not a formal assessment, educating the pilot and aviation workforce about mental health conditions can also assist in the detection of problems. This also can help to reduce social stigma associated with mental health problems and also alert crew to signs that may be of concern in fellow aviators.

The specific risk assessment is normally completed by a mental state evaluation. The examiner may also enquire about suicidal thoughts or plans that the individual may have or, indeed, a history thereof. Misuse of alcohol or recreational drugs and engaging in risky or sensation-seeking behaviors are a further line of enquiry. The clinical presentation of an individual who may be preoccupied, agitated or slowed in their behaviors may signal the presence of some risk but this is highly unlikely in the medical examiner's assessment interview. Feelings of hopelessness, despair, depression and self-neglect may be further indicators of risk. Finally, abuse or harm of the individual from a third party may signal that that individual is at risk of self-harm. It is, therefore, incumbent upon health care workers in the aviation industry, as well as pilots themselves, co-pilots and family members around them, to identify and signal risk in an individual who can then be referred for a specialist assessment and support (27, 28).

## Discussion

Self-harm history and risk for future suicidal attempts is relevant to the mental health assessment of pilots. In the recent anonymous web-based survey, based on PHQ-9 completed by 1,866 international airline pilots, 10 pilots reported thinking they would be better off dead or had thoughts of self-harm (38). This figure represents about (10/1,866) 0.5% of the pilots who participated in the survey. Self-harm ideation is not necessarily a prediction that the pilot would in real life plan to self-harm, however it needs to be remembered that self-harm is a strong predictor of suicide (14).

The complex association of self-harm and the impact of the COVID-19 pandemic has been intensively studied (13–16). A large registry study of the French national database *Programme de Médicalisation des Systémes d'Information* (PMSI) involved 53,582 self-harm hospitalisations during January–August 2020 (39). In this study, the decrease in cases of self-harm started at the same time when in the middle of March, a lockdown was enforced in France. This study provides clear evidence that COVID-19 policy can impact self-harm cases. Similar findings were also confirmed in the UK (40). Some theories suggest that lockdown may create a “pulling-together effect” (41). But it is also possible that there is reporting bias because patients may not visit health facilities and specialists due to pandemic restrictions and also a concern that their issues are not as pressing as other medical conditions (14). It is also possible that when the pandemic crisis finally ends, self-harm and suicide rates may increase (19).

What is then reported of those very few pilots who have carried out suicide by aircraft and their previous history of self-harm? Health history based on periodic medical examinations may be limited in accident investigation reports because only part of the relevant health information is self-reported in these examinations (37). Based on the limited history available in the National Transportation Safety Board (NTSB) reports self-harm did not precede aircraft assisted suicides (42–44).

We recommend that clinical risk assessment is taken into account, in addition to differential diagnosis. Also, the pilot's unique and specific impact of work stressors, including pandemic effects, needs to be taken into account in any pilot assessment. Evidence-based self-harm and suicide prevention strategies could be applied to aviation medicine and would need to be based on fluent access to aero-medical healthcare, strengthening treatment of mental disorders and chain of care without unnecessary delays (19).

## Author Contributions

Both authors listed have made a substantial, direct and intellectual contribution to the work, and approved it for publication.

## Conflict of Interest

The authors declare that the research was conducted in the absence of any commercial or financial relationships that could be construed as a potential conflict of interest.

## Publisher's Note

All claims expressed in this article are solely those of the authors and do not necessarily represent those of their affiliated organizations, or those of the publisher, the editors and the reviewers. Any product that may be evaluated in this article, or claim that may be made by its manufacturer, is not guaranteed or endorsed by the publisher.
